# Microstructure-Dependent Rotational Wear of Dental Glass-Ceramics Under Low Humidity

**DOI:** 10.3390/jfb17040204

**Published:** 2026-04-20

**Authors:** Estíbaliz Sánchez-González, Fernando Rodríguez-Rojas, Oscar Borrero-López

**Affiliations:** 1 Departamento de Ingeniería Mecánica, Energética y de los Materiales, Universidad de Extremadura, 06006 Badajoz, Spain; 2 Departamento de Ingeniería Mecánica, Universidad de Salamanca, 37700 Bejar, Spain

**Keywords:** dental prosthesis, dental glass-ceramics, microstructure, rotational forces, wear, bruxism, xerostomia

## Abstract

**Background**: The wear resistance of modern commercial glass-ceramic materials used in dental prostheses was investigated under cyclic contact conditions that included a rotational component. This loading mode has been largely overlooked in conventional in vitro wear testing, yet may be clinically relevant in patients with parafunctional conditions such as bruxism. **Methods**: Rotational loading was applied using an all-electric testing machine equipped with a biaxial actuator. Loading cycles combined a normal load (50 N) and a rotation (30°), at a frequency of 1 Hz. Microstructure and damage were characterized using advanced microscopy. **Results**: Rotational loading induced substantial damage across this class of materials, including the formation of glassy tribolayers with limited protective capability under the low-humidity conditions examined. Significant microstructure-dependent variations in wear volume were observed, with specific wear rates indicating severe wear (SWR above 10^−6^ mm^3^/N·m threshold) in three of the five materials tested. Lithium disilicate glass-ceramics, characterized by a high fraction of elongated reinforcement crystals, exhibited the greatest resistance to damage, whereas leucite-based glass-ceramics showed the lowest. The dominant wear mechanisms were plastic-deformation-induced grooving and fracture-driven chipping. The findings are interpreted within established wear models for brittle materials (Archard and fracture-based) and supported by numerical simulations of stress fields across multiple length scales. **Implications**: The results provide mechanistic insight into rotational wear damage in glass-ceramic systems, a material class particularly susceptible to such loading, and inform strategies for material selection and microstructural design aimed at improving prosthetic durability.

## 1. Introduction

Ceramic materials have long been widely used in dentistry due to their chemical stability, biocompatibility, and aesthetic properties. With the advent of computer-aided design/computer-aided manufacturing (CAD/CAM) technologies, which have mitigated some of the machining limitations associated with ceramic brittleness, ceramic-based materials have become the standard for indirect restorations such as crowns and veneers [1].

Glass-ceramics with biphasic microstructures (e.g., feldspathic, leucite-reinforced, and lithium-based systems), which offer superior aesthetics but lower strength—typically on the order of hundreds of MPa, compared to values exceeding 1000 MPa for zirconia [2]—are employed in restorations subjected to moderate loads. In contrast, zirconia-based ceramics, characterized by polycrystalline duplex microstructures and lower translucency but higher strength and toughness, are preferred for load-bearing and high-friction applications [3]. The clinical relevance of these materials is underscored by the substantial global markets (prosthetics) for dental zirconia [4] and dental glass-ceramics [5]. Furthermore, ceramics are compatible with additive manufacturing technologies, including 3D printing, which are poised to play a transformative role in next-generation dental prosthetics [6,7].

Despite these advantages, the relatively limited fracture toughness and wear resistance of dental ceramics continue to constrain prosthesis longevity, as documented in clinical studies [8,9]. This limitation is particularly pronounced in glass-ceramics, owing to the intrinsic brittleness of the glass matrix and the mechanical weakness of crystal–matrix interfaces. Compared to zirconia, glass-ceramics generally exhibit lower fracture strength [2,10], fracture toughness [10,11,12], resistance to chipping [13,14,15] and long-term durability (resistance to wear and fatigue) [12,16]. Moreover, significant performance differences exist among glass-ceramic systems as a function of microstructural architecture [2,11,17,18].

Although the fracture and wear of dental ceramics have been extensively investigated, continued advances in material design [6,7]—particularly the development of hierarchical microstructures across multiple length scales [19]—and in experimental methodologies sustain the need for renewed study. In recent work [20], decoupled biaxial actuators were employed to investigate in vitro the effects of rotational contact, a loading mode largely overlooked in conventional dental wear testing. Rotational forces can arise during mastication [21,22], as suggested by helicoidal and arc-like microwear markings reported in archeological/anthropological records [23], and may be further amplified under parafunctional conditions such as eccentric clenching bruxism [24].

Our prior study demonstrated that although rotational loading induces less wear than purely sliding contact, it can nevertheless produce substantial damage with potential strength-degrading consequences. In particular, biphasic glass-ceramics exhibited wear levels comparable to those observed in significantly softer polymer–ceramic composites. Key mechanical and microstructural parameters governing degradation under rotational loading were identified, with implications extending beyond dentistry to broader biological and anthropological contexts.

The present follow-up study focuses specifically on glass-ceramics, the most vulnerable material class identified previously, to assess specific microstructural effects such as the composition of the matrix and reinforcing crystals, as well as the content and morphology of the reinforcing phase. Experiments were conducted under ambient humidity conditions, representing physiological environments associated with reduced lubrication, such as xerostomia (dry mouth) arising from genetic predisposition or as an increasingly common side effect of medical treatments [25]. The experimental findings are interpreted within the framework of contact mechanics to derive microstructural design guidelines aimed at improving resistance to rotational and other complex contact modes. Ultimately, this work seeks to inform the development of more robust and durable glass-ceramic dental prostheses.

## 2. Materials and Methods

Plane parallel test specimens (3 per material) of surface area 1.2–1.5 cm × 1.2–1.5 cm and thickness slightly over 5 mm were cut by mechanical sawing from as-received dental ceramic CAD/CAM blocks, procured from commercial suppliers. In some cases, subsequent heating was conducted in a furnace (Multimat Touch, Dentsply Sirona, Charlotte, NC, USA) in a dental/prosthetic facility (Clínica David Maestre, Valverde de Leganés, and LAB Dental, Badajoz, Spain), following the manufacturer’s specifications.

Feldspathic glass-ceramic (F) (Vitablocs Mark II, Zahnfabrik H.Rauter GmbH & Co.KG, Bad Säckingen, Germany) and leucite-based glass-ceramic (L) (IPS Empress CAD, Ivoclar Vivadent, Schaan, Liechtenstein) samples were prepared from as-received CAD/CAM blocks (already fully dense).

Lithium disilicate (LD) samples (IPS emax.CAD, Ivoclar Vivadent, NY, USA) were crystallized in a vacuum atmosphere using the cycle: heating to 400 °C, soaking 6 min; heating to 820 °C, soaking 10 min; and heating to 840 °C, soaking 7 min. Cooling was conducted slowly (chamber closed) until 550 °C, and then quickly by opening the furnace doors.

Two types of lithia-zirconia glass-ceramics (ZLS) were used: ZLS1 (Celtra Duo, Dentsply Sirona, Charlotte, NC, USA) was employed as-received (already crystallized), while ZLS2 (Suprinity, Zahnfabrik H.Rauter GmbH & Co.KG, Bad Säckingen, Germany) specimens were crystallized in a vacuum atmosphere: heating to 400 °C, soaking 4 min; heating to 840 °C, soaking 8 min. Cooling was conducted slowly (chamber closed) until 680 °C, and then quickly by opening the furnace doors.

The specimen surfaces for mechanical testing and microscopic observation were prepared by lapping (30 μm), and subsequent polishing in an automatic polishing machine (Automet, Buehler, Lake Bluff, IL, USA) with diamond suspensions delivered from spray bottles (MetaDi, Buehler, Lake Bluff, IL, USA), using a routine consisting of the following steps: 15 μm (10 min), 9 μm (10 min), 6 μm (10 min), 3 μm (15 min), and 1 μm (20 min). During lapping and polishing, the applied force was 5 N, and the rotation speed of the supporting plate was 125 rpm. At the end of the surface preparation procedures, the final sample thickness was 5 mm.

Observations of the materials’ microstructure and damage were conducted in a scanning electron microscope (FE-SEM; Quanta 3D FEG, FEI, Eindhoven, The Netherlands) using secondary and backscattered electrons at accelerating voltages between 10 and 30 kV. The specimens were observed both uncoated and sputter gold-coated, at working distances between 5 and 12 mm. An image analysis of select SEM micrographs was conducted using the software Image-Pro Plus 6.0 (Media Cybernetics Inc., Rockville, MD, USA).

Mechanical properties were measured by Vickers indentation experiments (MV-1, Matsuzawa, Toshima, Japan) at room temperature in air. The tests were performed with a diamond tip, using a maximum load of *P* = 9.8 N (10 indentations/material). Hardness (*H*) and fracture toughness (*K_C_*) were estimated from the dimensions of the indentation scars, as measured by optical microscopy (Epiphot 300, Nikon, Tokyo, Japan) and image analysis software, using standard formulae [26,27,28]:

(1) $$H=\frac{2P}{d^{2}}$$

(2) $$K_{C}=0.016{\left(\frac{E}{H}\right)}^{1/2}\frac{P}{c^{3/2}}$$

where *d* is the length of the scar diagonal, *c* is the length of radial cracks, and *E* is the elastic modulus of the material.

**Figure 1:**
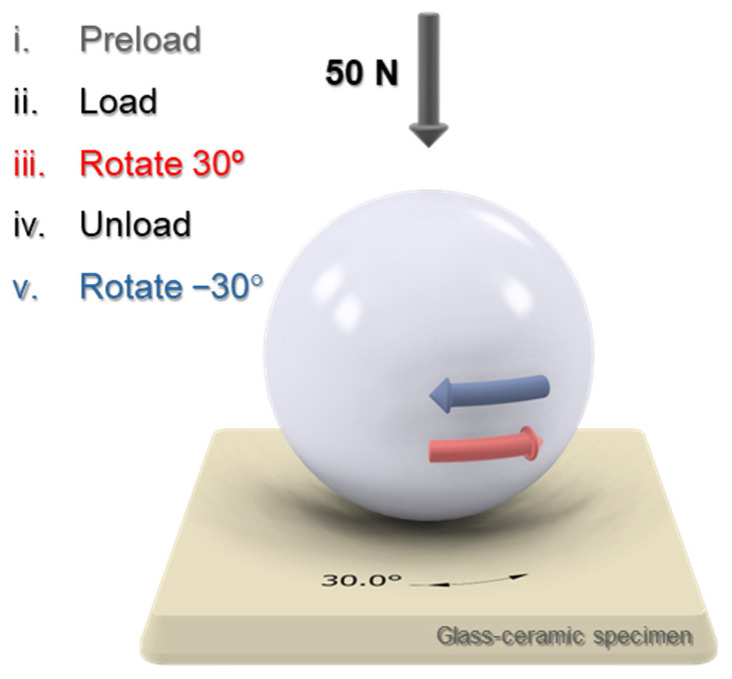
A schematic of the rotational tests performed, illustrating the different steps in each cycle. The rotational component is highlighted in red.

Rotational contact tests were conducted using an all-electric test machine equipped with a 1 kN load cell (Electropuls E10000; Instron, Canton, MA, USA). The load was applied via a mirror-finished yttria-stabilized zirconia sphere of diameter 12.7 mm (Goodfellow, Cambridge, UK). Zirconia was selected as the counterbody material because it is chemically inert and, being the hardest dental material, does not undergo significant damage during testing, thereby enabling isolation of the damage mechanisms in the glass-ceramic specimens. Each loading cycle comprised the following sequence (Figure 1): (i) application of a 3 N normal preload; (ii) increase to a target normal load of 50 N; (iii) rotation of the sphere by +30° under constant normal load; (iv) unloading; and (v) reverse rotation of −30° to the initial position under unloaded conditions. All five steps were performed with equal duration. The cycling frequency was 1 Hz. For each material, tests were performed for 10^4^, 5·10^4^ and 10^5^ contact cycles (at 1 Hz). The loading conditions were consistent with previous in vitro tribomechanical studies and produced Hertzian contact dimensions comparable to those in natural cuspal contacts [29]. Three independent rotational tests were conducted per material at each cycle count. Due to the technical limitations of the all-electric equipment required to conduct the large number of cycles programmed, which prevented the use of external lubrication, the tests were conducted in ambient conditions of temperature (19–24 °C) and humidity (40–60% relative humidity). Such test conditions were thus closer to extreme oral conditions (e.g., xerostomia). Although the experimental conditions did not fully replicate clinical conditions, they were identical across all tests and therefore enabled meaningful comparisons between materials. In particular, they allowed the assessment of subtle microstructural effects on resistance to damage under rotational contact loading, which is of clinical relevance.

Three-dimensional images of the wear scars were obtained by confocal microscopy (Leica DCM8, Leica Microsystems, Wetzlar, Germany; LeicaSCAN DCM8 v. 6.6.9.1). The scanned areas were levelled using the instrument software to define a reference plane prior to analysis. Assuming near-spherical cap geometry, wear volume was estimated using:

(3) $$V=\frac{\pi h}{6}(3a^{2}+h^{2})$$

where *a* is the scar radius and *h* is the scar depth. For each scar, *a* and *h* were measured from two independent two-dimensional profiles passing through the centre of the scar (i.e., two measurements of *V* per test). Since each condition was repeated three times, this resulted in a total of six measurements per material and number of cycles. Student’s *t*-tests were conducted on the wear volume data, with *p* < 0.05 considered to indicate statistically significant differences.

Relevant components of the contact stress field were simulated using the commercial software package FilmDoctor^®^ v16 (SIO^®^, Saxonian Institute of Surface Mechanics, Ruegen, Germany). The software calculates the elastic stress field from the contact conditions and the materials’ elastic property values, using the extended Hertzian model [30]. The model assumes fully elastic contact. While inelastic processes are also expected under rotational loading, glass-ceramic materials are predominantly brittle, and the elastic response is therefore expected to dominate. In any case, the simulated stress fields are used only qualitatively to provide insight into the damage mechanisms, rather than for quantitative predictions.

## 3. Results

**Figure 2:**
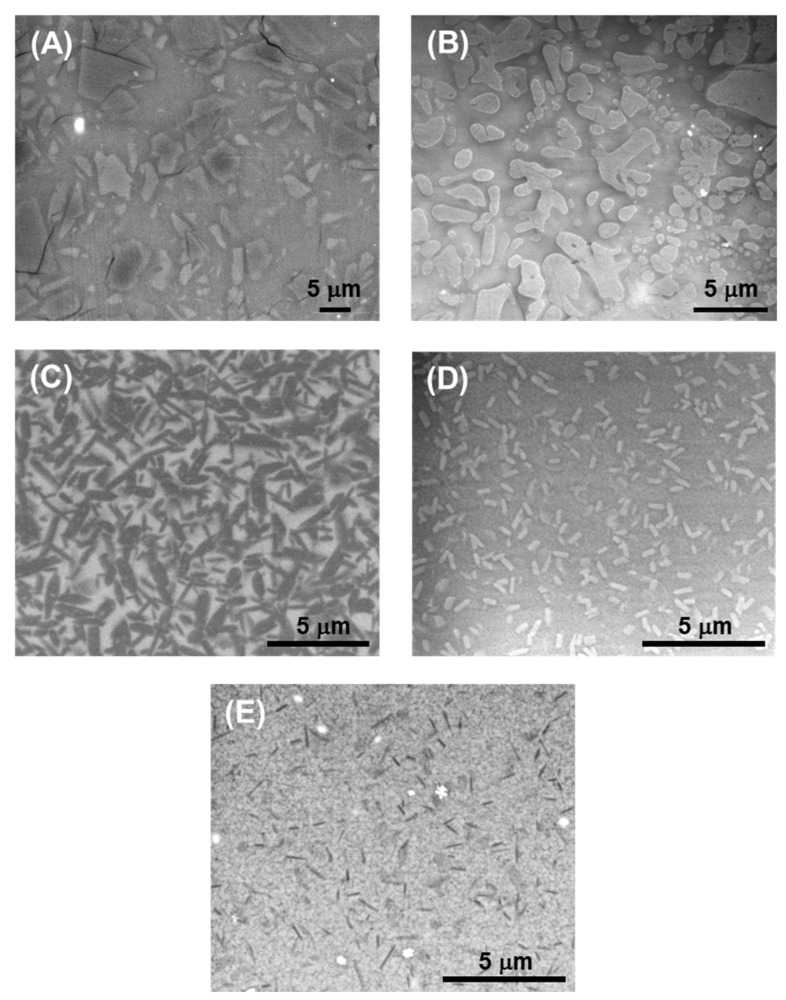
Scanning electron microscopy (SEM) images representative of the microstructure of the glass-ceramics investigated: (**A**) feldspathic, F; (**B**) leucite, L; (**C**) lithium disilicate, LD; (**D**) zirconia-lithia 1, ZLS1; and (**E**) zirconia-lithia 2, ZLS2.

Figure 2 presents representative SEM images (magnification range 5k–20k) of the microstructures of the glass-ceramic materials examined in this study, prior to testing. The microstructural features of these systems have been extensively described in previous work [17,31,32], but a concise summary derived from the observations in Figure 2 is provided here for completeness. All five materials exhibit a multiphase microarchitecture composed of a continuous glassy matrix containing a substantial population of crystalline phases. Accordingly, the differences among them arise primarily from variations in matrix chemistry and in the type, morphology, volume fraction, and dimensions of the embedded crystals.

In the F and L materials, the matrix consists of an aluminosilicate glass, whereas in the lithium-based materials it is a silicate (LD) or silicate/zirconosilicate (ZLS1 and ZLS2) glass. The reinforcing crystals (phase contents are reported as dimensionless quantities, expressed as fractions or percentages) correspond to feldspar (F, 40–45 vol%), leucite (L, 40–45 vol%), a mixture of lithium disilicate and phosphate (LD, 70 vol%), and a mixture of lithium silicates and phosphate (ZLS1, 50 vol%; ZLS2, 57 vol%).

Crystal morphology also varies markedly across compositions. F contains mostly equiaxed, micrometric crystals (typically ~5 µm, with occasional grains exceeding 10 µm). LD is characterized by elongated, rod-like crystals of similar scale (average ~2.2 µm). Both L and the ZLSs present bimodal crystal populations. In L, leucite crystals may develop partial dendritic outlines, producing a combination of equiaxed and elongated grains with mean sizes around 0.7 µm and 4 µm, respectively. The ZLSs contain elongated crystals averaging 0.7 µm, together with nanometric-equiaxed crystallites dispersed within the glass.

Table 1 summarizes the mechanical properties of the materials. All exhibit similar hardness values in the range of 5.2–6.2 GPa. These differences are not strongly correlated with crystal fraction; rather, the slightly higher hardness of the ZLS materials is attributed to the increased hardness of their zirconium-containing glass matrix [31]. Fracture toughness values are modest for all compositions, consistent with their predominantly brittle response. LD displays somewhat higher toughness, which is generally attributed to the presence of its elongated lithium disilicate crystals that promote crack-deflection and crack-bridging mechanisms [27,28]. Although the ZLS materials also contain elongated crystals, their lower volume fraction and smaller size render them less effective in contributing to energy dissipation upon crack opening. In addition, the zirconium-containing glass matrix of the ZLSs tends to be intrinsically stiffer and more brittle, further limiting their overall toughness.

**Figure 3:**
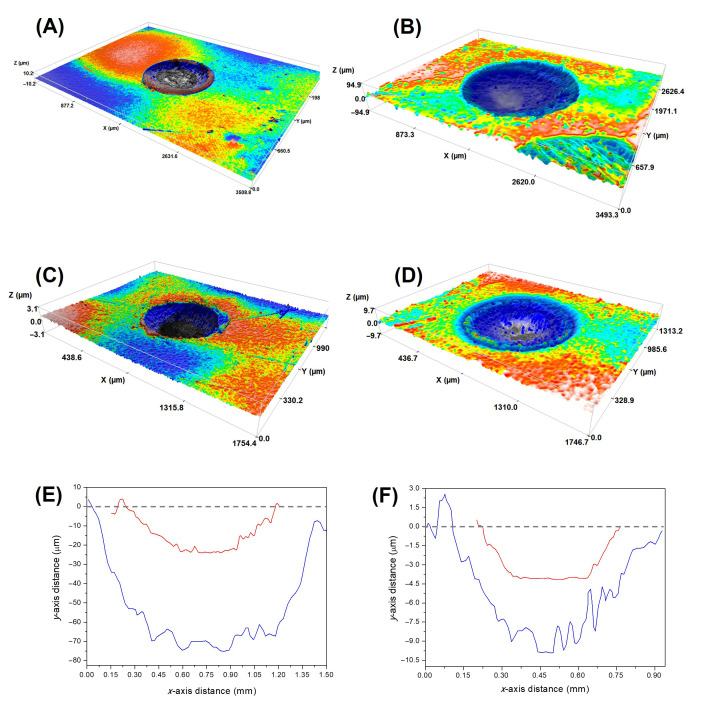
Representative confocal microscopy images of the surface scars on select materials: 3D images of (**A**) L, after 10^4^ cycles; (**B**) L, after 10^5^ cycles; (**C**) LD, after 10^4^ cycles; and (**D**) LD, after 10^5^ cycles. Sample 2D profiles across the centre are shown in (**E**) L glass-ceramic after 10^4^ cycles (red line), and 10^5^ cycles (blue line); and (**F**) LD glass-ceramic after 10^4^ cycles (red line), and 10^5^ cycles (blue line).

Figure 3 shows representative, low magnification 3D images of the scars obtained at the end of the rotational tests. The prolonged rotational contact caused significant wear to all the glass-ceramics, with scar depths greater than those predicted by the Hertzian contact theory for purely axial loading at the same normal load [33]. Moreover, even after surface cleaning prior to microscopy imaging, debris can be observed near the scar, which provides additional evidence of the occurrence of material removal processes. For a given material, both scar diameter and depth increase with the number of contact cycles. Comparatively, after 10^5^ cycles the diameter and depth of the wear scar are smaller in LD and F, and larger in the L and ZLS2 materials.

**Figure 4:**
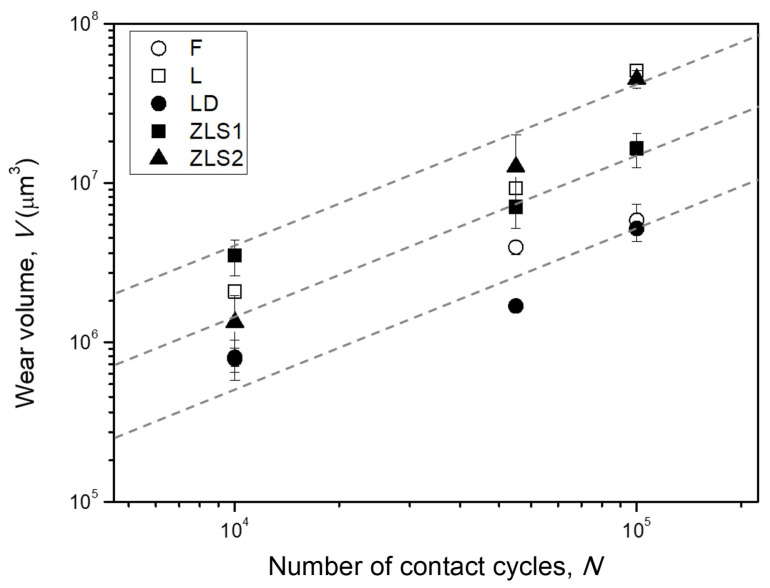
The wear volume of the dental glass-ceramics as a function of the number of rotational cycles. Each symbol and error bar correspond, respectively, to the mean value and standard deviation of the experimental measurements (a total of six per material/number of cycles). The dashed, grey lines correspond to the reference lines of slope 1 in the double-log scale.

Figure 4 shows, for all glass-ceramics, the average wear volumes measured from the confocal microscopy images at each cycle count. As expected, wear volume increases progressively with the number of contact cycles. Although the overall trends (i.e., the rates of increase) are broadly comparable across materials, the absolute wear volumes differ, reflecting variations in microstructure. LD and F show the lowest wear, followed by ZLS1, while L and ZLS2 show the most pronounced material loss.

**Figure 5:**
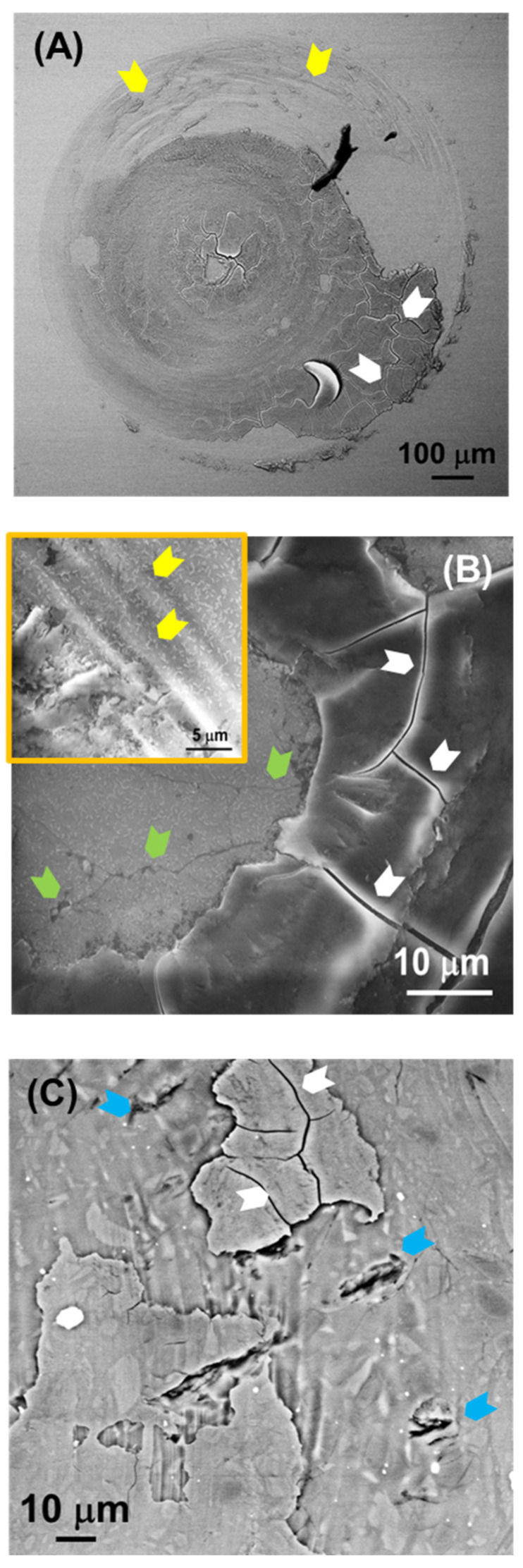
SEM images of the wear scar on the ZLS1 and F materials after 5·10^4^ cycles: (**A**) ZLS1, low magnification (macro-contact); (**B**) ZLS1, intermediate mag. (inset shows detail at high mag.); and (**C**) F, intermediate mag. The white, yellow, green and blue arrows highlight, respectively, examples of macrocracks, plastic grooves, microcracks and pits. The images are reformatted from a preceding study [20].

**Figure 6:**
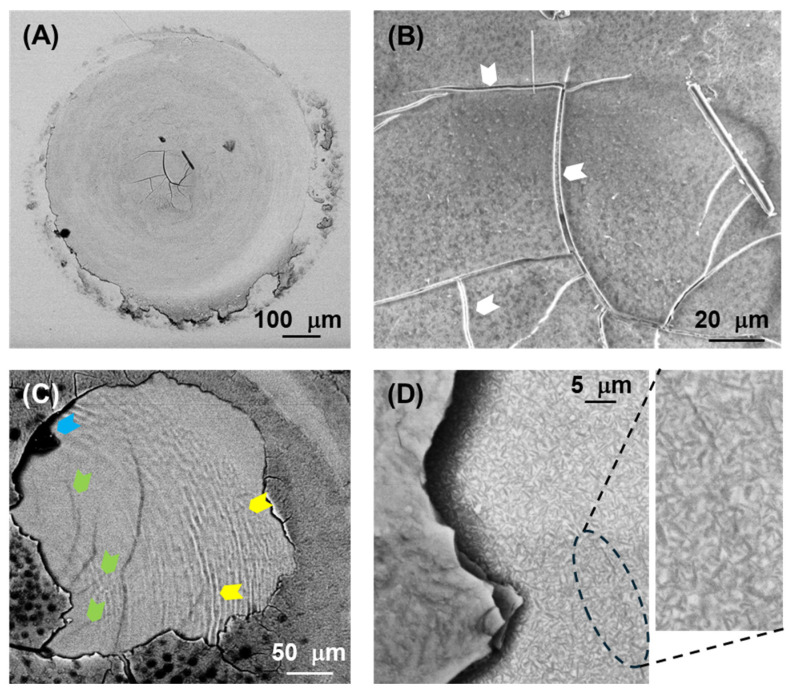
SEM images at different magnifications representative of the rotational wear scar on the surface of the LD glass-ceramic after 5·10^4^ contact cycles: (**A**) low magnification (macro-contact); (**B**,**C**) intermediate magnification; and (**D**) high magnification. The white, yellow, green and blue arrows highlight, respectively, examples of macrocracks, plastic grooves, microcracks and pits.

**Figure 7:**
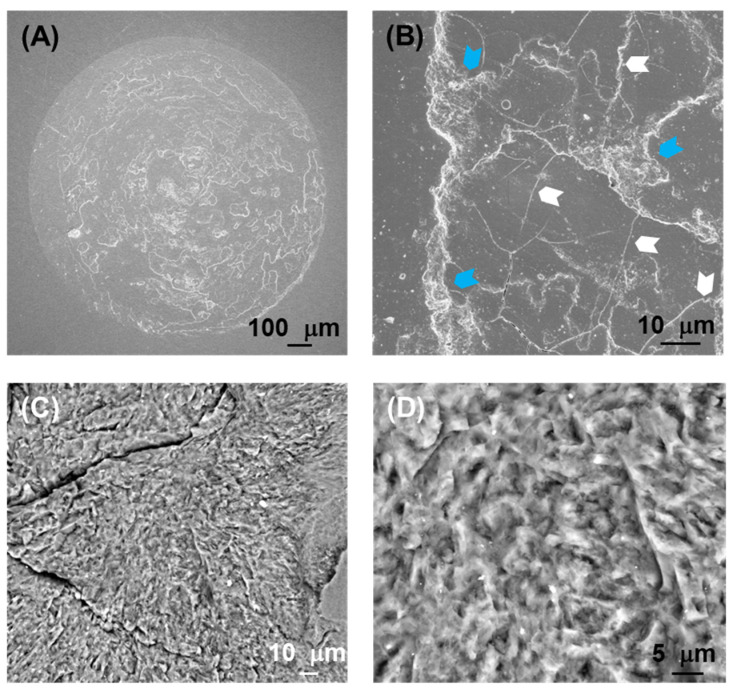
SEM images at different magnifications representative of the rotational wear scar on the surface of the L glass-ceramic after 5·10^4^ contact cycles: (**A**) low magnification (macro-contact); (**B**,**C**) intermediate magnification; and (**D**) high magnification. The white and blue arrows highlight, respectively, examples of macrocracks and pits.

**Figure 8:**
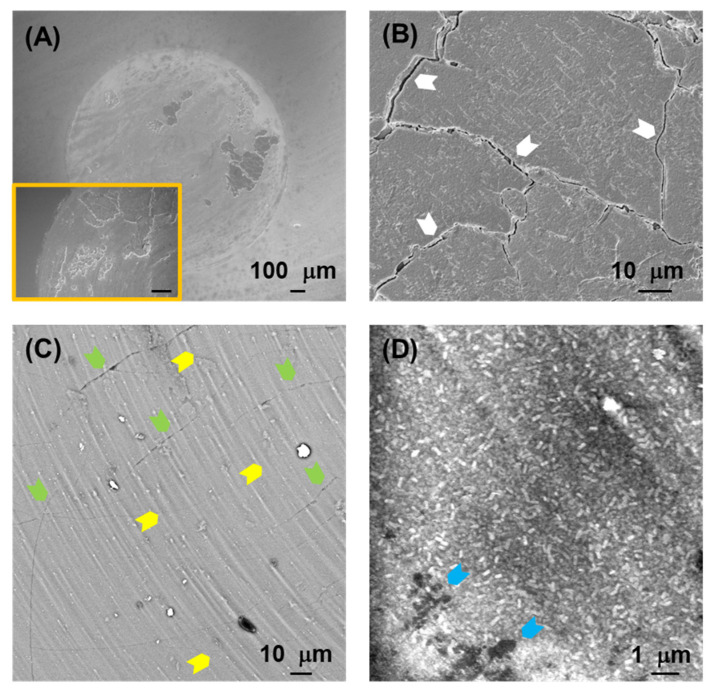
SEM images at different magnifications representative of the rotational wear scar on the surface of the ZLS2 glass-ceramic after 5·10^4^ contact cycles: (**A**) low magnification (macro-contact), with inset showing further details of chipped zones; (**B**,**C**) intermediate magnification; and (**D**) high magnification. The white, yellow, green and blue arrows highlight, respectively, examples of macrocracks, plastic grooves, microcracks and pits.

Figure 5, Figure 6, Figure 7 and Figure 8 show SEM images representative of the damage induced by the rotational contacts at the relevant length scales. Figure 5 summarizes the observations in the F and ZLS1 materials from our previous study [20], while Figure 6, Figure 7 and Figure 8 present in greater detail the damage observed in the other three dental glass-ceramics investigated in the present work. The new observations confirm that all dental glass-ceramics form tribolayers under cyclic rotational contact in ambient conditions, without external lubrication. These tribolayers can cover a substantial portion of the wear scars and, from a mechanical perspective, are fundamentally brittle in all cases, as indicated by the numerous cracks present on their surfaces (examples are highlighted by white arrows). Whereas in some glass-ceramics (e.g., L and the ZLSs) the tribolayers appear relatively homogeneous, in others—most notably LD—they contain submicron-sized inclusions.

The regions where the tribolayers have been pulled out reveal microwear markings in the form of circular grooves (examples are highlighted by yellow arrows), together with numerous microcracks (green arrows) and occasional scattered micron-sized pits (blue arrows). Higher-magnification images (Figure 5B,C, Figure 6C, Figure 7C and Figure 8D) illustrate the extent of these microwear markings relative to the size of the reinforcing crystals in each material. The interaction between the various damage modes and the microstructure is most pronounced in the two extremes in Figure 4 (LD and L). LD is the only material in which the relatively large, elongated crystals are able to deflect microcracks (e.g., Figure 6D). In contrast, L is the only glass-ceramic whose wear scar appears rough at higher magnifications, with fracture surfaces clearly revealing the contours of the dendritic leucite crystals (Figure 7C,D).

## 4. Discussion

Despite the evidence of curved microwear markings in the archeological/anthropological record [21,23], most experimental studies of fracture and wear in dental materials have traditionally neglected the effects of the rotational forces responsible for such features. To evaluate the influence of rotational components on contact damage in modern dental glass-ceramics—a material class particularly susceptible to such degradation—a decoupled biaxial loading configuration was employed that permits the controlled superposition of motion modes. The imposed rotation amplitude of 30° was intentionally set above physiological values, not to replicate mastication, but to amplify damage signatures and clarify underlying mechanisms. Loading conditions were therefore selected to prioritize repeatability and sensitivity over strict physiological realism. Accordingly, the experimental protocol represents not a chewing simulator, but a reductionist test platform designed for the comparative assessment of material responses under well-defined conditions. In addition, the ambient humidity conditions employed do not replicate the fully lubricated oral environment, and therefore direct clinical extrapolation should be made with caution. Viewed within this framework, the results obtained demonstrate that (i) rotational forces can induce substantial damage, and (ii) resistance to contact damage and attendant wear volume can vary by up to an order of magnitude among aesthetically and mechanically (i.e., elastic modulus, hardness and fracture toughness) very similar materials, highlighting the governing role of microstructural architecture.

For all materials, the wear volume increases with the increasing number of contact cycles, approximately following a linear trend of slope 1 in a double-log plot (dashed lines in Figure 4), in compliance with a classical Archard-type relationship:

(4) $$V=K\frac{FL}{H}$$

where *K* is a material-specific wear coefficient, *F* is the applied normal load, *H* the material hardness, and *L* the equivalent sliding distance, which is proportional to the number of contact cycles [20]. After 10^5^ contact cycles, the wear volumes ranged between 0.051 mm^3^ (L) and 0.005 mm^3^ (LD). The statistical analysis indicates that the differences between LD and F (*p* = 0.61), and between ZLS2 and L (*p* = 0.27) are not significant. The differences between LD/F and ZLS1 (*p* ≤ 0.03) and between ZLS2/L and ZLS1 (*p* ≤ 0.002) are significant.

The measured wear volumes correspond to sliding-equivalent specific wear rate (SWR) values of 2.3·10^−5^ mm^3^/N·m (L), 2.1·10^−5^ mm^3^/N·m (ZLS2), 1.1·10^−5^ mm^3^/N·m (ZLS1), 4.9·10^−6^ mm^3^/N·m (F) and 4.2·10^−6^ mm^3^/N·m (LD) [20]. Although such SWRs are lower than those reported for tests involving axial and lateral forces only, they remain non-negligible. In particular, the SWR values obtained for L, ZLS1, and ZLS2 clearly fall within regimes classified as severe wear for brittle materials, while those obtained for LD and F lie at the threshold (10^−6^ mm^3^/N·m [34]). While absolute wear rates may vary with different antagonist materials, the relative trends between materials are expected to remain consistent under comparable loading conditions. These findings indicate that, in situations where a rotational load component is likely—such as in patients exhibiting parafunctional activity—the potential contribution of rotational contact to prosthesis degradation should not be overlooked.

The pronounced disparity in SWR values among the various glass-ceramics—approaching one order of magnitude—can be rationalized in terms of the microstructural dependence of the dominant material removal mechanisms. An examination of the wear surfaces after testing (Figure 5, Figure 6, Figure 7 and Figure 8) reveals that abrasion is the prevailing wear mode across all materials. As is typical in the abrasive wear of brittle solids [35], the operative mechanisms comprise a combination of plastic deformation and fracture.

**Figure 9:**
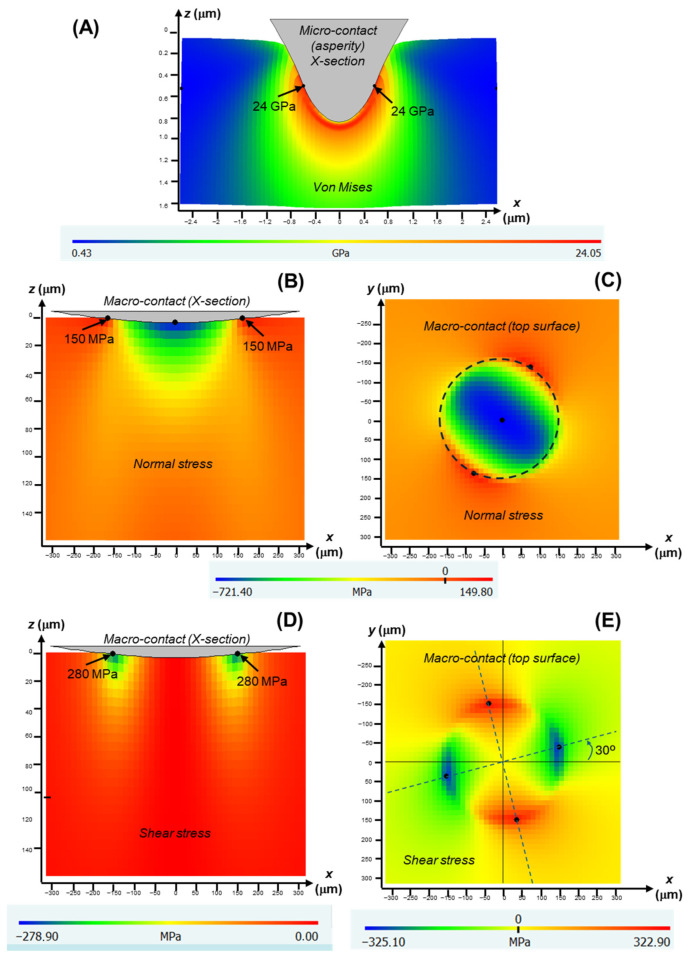
FilmDoctor^®^ simulations of relevant components of the stress field induced on a model isotropic, elastic layer of elastic modulus 73 GPa by a rotational contact (normal load 50 N, torque 5 N) applied with a zirconia sphere. (**A**) Von Mises stress generated by an asperity microcontact (radius 1 μm, resolved load 50 mN, torque 5 mN [20]); cross-sectional image. Normal stress in *x*-axis direction (σ*_xx_*): macro-contact (**B**) cross-section; and (**C**) surface, with dashed line marking the contours of the contact circle. Shear stress (τ*_xy_*): macro-contact (**D**) cross-section and (**E**) surface. Plots in cartesian coordinates, with *z* normal loading direction.

Plastic deformation is evidenced by circular grooves/scratches, typically of submicron width, aligned with the direction of sphere rotation (the yellow arrows in Figure 5, Figure 6, Figure 7 and Figure 8). It is caused by large shear stresses generated beneath sharp asperity microcontacts. As illustrated in Figure 9A, the numerical simulations performed for a model isotropic material with an elastic modulus of 73 GPa (somewhere in the middle of the modulus range of the investigated glass-ceramics) predict substantial subsurface shear stresses (~GPa) concentrated near the contact interface. It should be noted that the simulations are intended to provide qualitative and comparative insight into the stress fields, rather than to yield exact quantitative predictions of critical stress values.

Fracture is evidenced by the numerous cracks observed on the wear surfaces (the white and green arrows in Figure 5, Figure 6, Figure 7 and Figure 8). These cracks are propagated by tensile stresses generated during the contact. As shown in Figure 9B,C, significant tensile stresses develop near the periphery of the macro-contact, where crack initiation is most likely. Progressive crack propagation and coalescence ultimately result in the dislodgement of relatively large fragments through chipping. Because chipped/spalled particles can reach dimensions of several micrometres, fracture-driven material removal contributes more substantially to overall wear volume than plastic-deformation-induced grooving.

In addition, under the ambient humidity conditions tested, all glass-ceramics exhibited evidence of tribolayer formation. It is hypothesized that the development of these layers is due to frictional heating at contacting asperities, including both surface asperities and third-body particles entrapped within the contact, which are thermally sensitive due to their high surface-to-volume ratio. Given that contacting asperities are highly localized and temperature rises at microcontacts are transient and short-lived, the direct experimental measurement of flash temperatures is extremely challenging, and modelling approaches are therefore commonly employed. Established flash-temperature models in tribology indicate that even at relatively low sliding speeds, frictional heating at microcontacts can generate transient temperatures up to an order of magnitude higher than those at the nominal macro-contact, with further increases predicted as asperity size decreases [36]. While these temperatures remain below the melting point of glass-ceramic materials, they may be sufficient to locally soften the residual glass phase or detached debris. The softened glass can subsequently undergo plastic flow and smearing under the substantial shear stresses generated during the rotational portion of the contact cycle. As an example, Figure 9 presents numerical simulations of the shear stress distribution produced by a model rotational contact, shown in cross-section (Figure 9D) and at the surface (Figure 9E). The highest shear stresses occur near the edges of the contact and extend several micrometres beneath the surface. This combination of localized thermal softening and high interfacial shear stress promotes the formation of glazed tribolayers that progressively grow with repeated contact cycles. Given the transient and metastable nature of flash processes, the chemical composition of the tribolayers is expected to be complex. Detailed characterization by techniques such as EDS or XRD would be required to elucidate these aspects, which is beyond the scope of the present work. Although tribolayers can, in some systems, provide a protective effect by reducing friction, the layers formed in these glass-ceramic materials are mechanically detrimental—owing to the intrinsic brittleness of glass, they are susceptible to cracking and fragmentation under the tensile components of the cyclic contact stress (Figure 9B,C).

Although the extent of damage may vary depending on the antagonist material, the damage mechanisms identified under the controlled conditions of the present study are expected to be representative of those occurring under similar loading and environmental conditions.

In general, a material’s resistance to plastic deformation is measured by its hardness, while the resistance to the propagation of cracks is measured by its fracture toughness. Indeed, established models of abrasive wear in ceramic materials, such as [37], based on contact-fracture mechanics, predict that wear volume scales as:

(5) $$V=k\frac{F^{5/3}L}{H^{1/2}K_{C}^{2/3}}\sim 1/H^{1/2}K_{C}^{2/3}$$

where *k* is a dimensionless coefficient [37].

**Figure 10:**
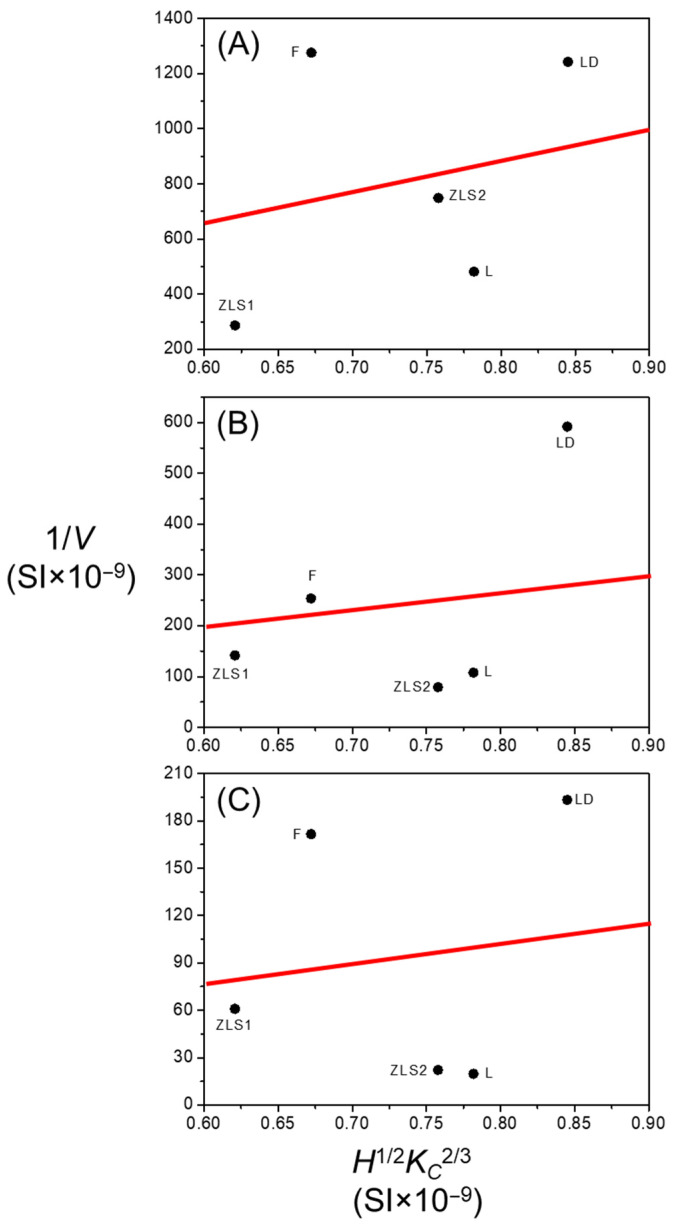
Scatter plots of resistance to wear, 1/*V*, as a function of the material parameter *H*^1/2^*K_C_*^2/3^ for the investigated dental glass-ceramics. The points correspond to experimental data from the rotational tests conducted at (**A**) 10^4^ cycles, (**B**) 5·10^4^ cycles, and (**C**) 10^5^ cycles. In each graph, the red line represents the best linear fit to the experimental data obtained using the least-squares method.

Accordingly, Figure 10 examines the correlation between the wear resistance (1/*V*) and the material parameter *H*^1/2^*K_C_*^2/3^. A reasonably strong correlation is observed at the earlier stages of the experiments (R^2^ = 0.8 at *N* = 10^4^ cycles), but the relationship progressively weakens with increasing contact cycles (R^2^ = 0.6 at *N* = 5·10^4^ cycles, R^2^ = 0.5 at *N* = 10^5^ cycles). This suggests that the effective contact conditions evolve during testing, likely due to the formation, modification, and removal of tribolayers with different mechanical properties. Experimental confirmation is hindered by the small thickness and high surface roughness of the tribolayer, which preclude the use of depth-sensing indentation techniques for reliable localized mechanical property measurements. Nevertheless, the contact-fracture mechanics framework, together with the simulated stress field (Figure 9), provides a sound theoretical basis for the comparative interpretation of the differences between materials as a function of microstructure.

The glass-ceramic most resistant to wear from rotational contacts is LD. This is attributed to the presence of reinforcing crystals of elongated shape in its microstructure. Such rod-like crystals are particularly effective in dissipating energy from propagating cracks by activating toughening mechanisms both ahead (crack deflection) and behind the tip (crack bridging) [27,28]. Consequently, LD displays a significantly higher *K_C_* value than the other glass-ceramics, resulting in reduced material removal by chipping. A clear contrast is observed between the glassy (highly brittle) tribolayer, where cracks propagate mostly in a straight line, and the bulk microstructure, where cracks follow more tortuous paths due to microstructural toughening (e.g., inset of Figure 6D). ZLS materials exhibit lower wear resistance than LD. Although their microstructures also contain elongated reinforcing crystals, both the size and volume fraction of these crystals are comparatively smaller than in LD, resulting in lower *K_C_* values (Table 1) due to less effective toughening [31]. As in LD, the (less effective) toughening mechanisms in the ZLSs operate primarily within the bulk material rather than within the more brittle glassy tribolayer.

Among the aluminosilicate-based glass-ceramics, material F achieves a wear resistance comparable to that of LD (the differences between LD and F are not statistically significant) despite exhibiting, a priori, less favourable microstructural features, including a relatively low content of crystals of large size and equiaxed shape, as well as comparatively lower *H* and *K_C_* values. This behaviour can be rationalized by its significantly lower elastic modulus, which reduces the magnitude of tensile stresses available to drive crack propagation under rotational contact [20]. As a result, cracking appears largely confined to the tribolayer. In contrast, material L exhibits the lowest resistance to rotational wear, despite having a similar elastic modulus than F, and higher values of *H* and *K_C_*. A plausible explanation is that the tribolayer formed on L adheres more strongly to the underlying bulk material than in the other glass-ceramics. This may be related to the dendritic morphology of its reinforcing crystals, which could promote greater interconnection and structural continuity across the tribolayer–bulk interface. Consequently, cracks propagating across the wear surface of L are less likely to deflect at a weak interface and instead penetrate deeper into the bulk material. This leads to the detachment of larger chip particles and the formation of rougher fracture surfaces, as observed in Figure 3E and Figure 7C,D. In contrast, spall particles generated in the other materials appear shallower and exhibit flatter detachment surfaces (e.g., Figure 3F, Figure 5, Figure 6 and Figure 8). The production of larger, rougher debris in L further enhances third-body abrasion, thereby exacerbating wear.

The results obtained in glass-ceramics must be interpreted within the broader framework of dental prosthetic materials, which also includes high-hardness materials such as zirconia and soft polymer-based composites, albeit with appropriate distinctions. In our previous work across multiple material classes, hardness and interfacial strength (i.e., short-crack toughness) emerged as primary indicators of resistance to rotational contact damage, with fine-grained zirconia exhibiting the highest overall resistance [20]. Within the currently investigated specific class of dental glass-ceramics, however, which are intrinsically more brittle, long-crack toughness appears to be the more relevant descriptor of resistance to rotational wear. Additional efforts are underway to extend the current methodology to characterize the rotational wear of human dental enamel. This remains challenging due to the complex anatomical geometry of natural teeth, which makes it difficult to obtain specimens of sufficient size and surface quality for controlled mechanical testing.

It should be emphasized that the present results are specific to low-humidity conditions (40–60% relative humidity), such as those simulated in this study. In contrast, the oral environment is characterized by near-saturated humidity and the presence of a saliva film with higher viscosity and complex rheological properties compared to water. As such, direct quantitative comparison between these conditions is not straightforward. Clinically, these findings are more relevant to individuals exhibiting parafunctional behaviours such as eccentric tooth clenching, which may amplify rotational loading components, as well as to patients affected by xerostomia (dry mouth). Under fully lubricated conditions, the formation of a tribolayer is unlikely. Indeed, previous in vitro sliding-wear studies have demonstrated that tribolayer development is suppressed in the presence of artificial saliva [16,31]. The tribolayers observed here are predominantly glassy in nature and, being more brittle than the underlying glass-ceramic bulk, do not confer a protective effect under cyclic contacts involving rotational forces, which generate large tensile stress on the surface.

The present findings carry important implications for the microstructural design of dental prostheses tailored to specific functional and clinical demands. In cases where rotational loading in low-humidity conditions is clinically relevant, enhancing wear resistance requires the optimization of both hardness and fracture toughness. In glass-ceramics, hardness may be increased, for example, by raising the volume fraction of reinforcing crystalline phases, while toughness can be improved through the incorporation of elongated crystals of sufficient size and aspect ratio to promote effective crack deflection and bridging. In addition, reducing the effective elastic modulus at the contact surface may mitigate tensile stress magnitudes and thereby limit crack propagation. This may be achieved through the tailored control of crystal content and modulus, or alternatively through the development of graded microstructures [38], as in natural enamel [39]. Such microstructural optimization inevitably involves trade-offs. Increasing crystal volume fraction and/or crystal size may adversely affect light scattering and translucency, potentially compromising the aesthetic appeal that constitutes a primary advantage of glass-ceramics. Therefore, future material design must balance mechanical durability against optical performance to achieve clinically optimal outcomes.

## 5. Conclusions

The present study investigated the contact damage and wear of dental glass-ceramics subjected to cyclic rotational loading against hard zirconia antagonists. The main conclusions are:

1. Rotational loading induces significant wear in dental glass-ceramics, with wear volumes varying by up to one order of magnitude as a function of microstructure.
2. The dominant wear mechanisms are plastic-deformation-induced grooving at microcontact asperities and fracture-driven chipping.
3. Under low-humidity conditions, prolonged rotational contact promotes the formation of brittle, mechanically unstable tribolayers.
4. Lithium disilicate exhibits the highest resistance to rotational damage, attributable to its comparatively greater fracture toughness associated with a high fraction of elongated crystals.
5. The findings have clinical relevance for parafunctional behaviours such as eccentric tooth clenching, as well as for patients affected by xerostomia (dry mouth).

## Figures and Tables

**Table 1:** The contact mechanical properties (elastic modulus, *E*; hardness, *H*; and fracture toughness, *K_C_*) of the dental glass-ceramics employed in this study.

	*E* (GPa)	*H* (GPa)	*K_C_* (MPa·m^1/2^)
Feldspathic, F	48 ^a^	5.2 ± 0.6	0.9 ± 0.2
Leucite, L	65 ^b^	5.8 ± 0.4	1.04 ± 0.06
Lithium disilicate, LD	88 ^c^	5.6 ± 0.1	1.20 ± 0.05
Lithia-zirconia, ZLS1	99 ^c^	6.2 ± 0.2	0.70 ± 0.03
Lithia-zirconia, ZLS2	104 ^b^	5.9 ± 0.4	0.98 ± 0.03

^a^ From [16]. ^b^ From [32]. ^c^ From [13].

## Data Availability

The original contributions presented in this study are included in the article. Further inquiries can be directed to the corresponding author.

## Abbreviations

The following abbreviations are used in this manuscript:

LD	Lithium disilicate glass-ceramics
ZLS	Lithia-zirconia glass-ceramics
F	Feldspathic glass-ceramics
L	Leucite glass-ceramics
*H*	Hardness
*K_C_*	Fracture toughness
*V*	Wear volume
SWR	Specific wear rate
